# [Retracted] Antiproliferation effect of the uremic toxin *para*-cresol on endothelial progenitor cells is related to its antioxidant activity

**DOI:** 10.3892/mmr.2025.13584

**Published:** 2025-06-02

**Authors:** Limin Pan, Xiaoting Ye, Jiguang Ding, Yu Zhou

Mol Med Rep 15: 2308–2312, 2017; DOI: 10.3892/mmr.2017.6230

Following the publication of this paper, it was drawn to the Editor's attention by a concerned reader that the ‘40 μg/ml *p*-cresol’ data panel in Fig. 2 on p. 2310 was strikingly similar to a data panel in a paper written by different authors at different research institutes that had already been published in the journal *PLoS One*.

In view of the fact that the abovementioned data had already apparently been published prior to its submission to *Molecular Medicine Reports*, the Editor has decided that this paper should be retracted from the Journal. The authors were asked for an explanation to account for these concerns, but the Editorial Office did not receive a satisfactory reply. The Editor apologizes to the readership for any inconvenience caused.

